# Carboxypeptidase A3 expression in canine mast cell tumors and tissue-resident mast cells

**DOI:** 10.1177/03009858211062636

**Published:** 2021-12-12

**Authors:** Sanna Hämäläinen, Lauri Kareinen, Antti Sukura, Ilona Kareinen

**Affiliations:** 1 University of Helsinki, Helsinki, Finland

**Keywords:** CPA3, dog, immunohistochemistry, intestine, liver, mast cell tumor, mastocytoma, skin, spleen

## Abstract

Mast cell tumors (MCTs) are one of the most common cutaneous malignancies in dogs. Previous studies have reported expression of mast cell–specific proteases chymase and tryptase in canine cutaneous MCTs and in connective tissue and mucosal mast cells. In humans and rodents, mast cells express an additional specific protease, carboxypeptidase A3 (CPA3). In this article, we describe CPA3 immunoreactivity in connective tissue, visceral, mucosal, and neoplastic mast cells in dogs. Positive immunolabeling for CPA3 was observed in nonneoplastic mast cells in 20/20 formalin-fixed paraffin-embedded normal tissues (skin, liver, spleen, intestine), and in 63/63 MCTs irrespective of their histological grade. CPA3 protein expression was comparable to that of c-kit in both the nonneoplastic and neoplastic mast cells. Three distinct labeling patterns (membranous, diffuse, and focal cytoplasmic) were observed for CPA3 in MCTs. The focal cytoplasmic labeling pattern was associated with high-grade MCTs staged with the Kiupel 2-tier grading criteria. We propose CPA3 as a novel immunohistochemical marker for canine mast cells in health and disease.

Mast cell tumors (MCTs) are one of the most common cutaneous tumors in dogs, and in a recent Swiss study, MCTs were reported as the most common cutaneous tumors representing approximately 16% of all skin tumors in dogs.^15^ Diagnosis of MCTs relies on cytological and histopathological examination of the cutaneous mass, and when needed, immunohistochemistry and histochemical stains can be used in differentiating MCTs from other round cell tumors. The most distinct morphological feature of mast cells is their high content of secretory granules that fill the cytoplasm and stain pale grey/blue with hematoxylin-eosin stain.^18^ The secretory granules contain large amounts of negatively charged proteoglycans which stain with cationic dyes, such as toluidine blue, and give the typical metachromatic staining pattern for mast cells.^47^ Mast cells express the stem cell factor receptor c-kit in every step of maturation and development,^11^ and immunohistochemistry for c-kit is probably the most common marker used in identifying MCTs. MCTs as well as cutaneous, cardiac, and intestinal mast cells in dogs contain the proteases tryptase and chymase^3,10,21,31,46,48^ in their secretory granules. Chymase and tryptase can be used as additional immunohistochemical markers both in differentiating canine mast cell subpopulations in tissues^20,46^ and in identifying MCTs.^8^

In addition to tryptase and chymase, mast cells in humans, mice, and rats express an additional mast cell–specific secretory protease, CPA3,^33^ also called MC-CPA. CPA3 expression is considered to be restricted to mast cell secretory granules, with the exception of reported CPA3 expression on RNA level in basophils of patients with allergic conditions.^25,37^ CPA3 belongs to the peptidase family M14 (carboxypeptidase A family), and similarly to pancreatic CPA, it is an exopeptidase cleaving C-terminal amino acid residues from proteins and peptides.^32^ CPA3 in humans is typically co-expressed in mast cells expressing both tryptase and chymase^33^ but is also expressed in only tryptase-positive mast cells associated with allergic diseases.^1,7^ In mice and rats, connective tissue mast cells express all 3 proteases (chymase, tryptase, and CPA3), while mucosal mast cells only express chymase.^2,33^ CPA3 in mast cells is stored in secretory granules and is tightly bound to negatively charged proteoglycans, especially heparin.^33^ CPA3 appears to be essential for secretory granule homeostasis. Mice lacking CPA3 do not have defects in mast cell function but present with a morphologically immature mast cell phenotype, and have an altered staining pattern compared with mast cells from wild type mice.^9^ Following exocytosis, CPA3 remains heparin-bound, and probably bides in the close vicinity of the degranulating mast cell.^34^ CPA3 is encoded by a single gene (*CPA3*) spanning over 32 kb and in humans and mice is located on chromosome 3 and in rats on chromosome 2.^34^ In dog the CPA3 gene is located on chromosome 23 (https://www.ncbi.nlm.nih.gov/gene/).

The true physiological role of CPA3 remains obscure. However, CPA3 has some distinct functions in innate immunity. CPA3 can deactivate snake venom (sarafotoxin) by degrading it, thus enhancing survival in experimental mouse models.^29,39^ Similarly, CPA3 can proteolytically modify endothelin-1 by removing its C-terminal Trp residue which protects against endothelin-1-induced lethality in a mouse model mimicking sepsis.^39^ CPA3 might also play a role in fighting against parasitic infections. *Ascaris suum* roundworms produce a CPA3 inhibitor to improve parasite survival during infection,^38^ and in humans *Strongyloides stercoralis* infection leads to increased serum CPA3 concentration,^42^ suggesting that this enzyme could play a role in *S. stercoralis* infection. Additionally, a variety of functional substrates for CPA3 have been recognized in vitro, including apolipoprotein B,^23^ neurotensin,^14,35^ and angiotensin I.^13^ The physiological relevance of these functions remains unknown.

To our knowledge no previous reports of CPA3 expression in canine mast cells exist. The objectives of this research were to study CPA3 protein expression in canine mast cells, both in nonneoplastic and neoplastic cells, and to evaluate cellular labeling patterns of CPA3 in MCTs and their associations to histological grading of the tumors.

## Materials and Methods

### Nonneoplastic Tissue Samples for Immunohistochemistry

Nonneoplastic canine mast cells were evaluated for their CPA3 tissue expression by immunohistochemistry. Tissues normally rich in mast cells (skin, small intestine)^24^ and tissues where mast cells are few but still a normal physiological feature (liver, spleen)^28,36^ were selected. Skin, liver, spleen, and small intestine (5 samples each) were obtained from the tissue archives of the University of Helsinki, Faculty of Veterinary Medicine, Pathology Unit. Samples originated from 10 necropsy cases, and due to lack of sufficient number of skin samples without pathological findings, of 1 skin biopsy case submitted to the Pathology Unit between June 2011 and May 2018 for diagnostic purposes (Suppl. Table S1). The cases were reviewed from the patient data system (ProvetNet, Finnish Net Solutions, Finland) with the inclusion criteria being that the selected tissue was diagnosed as “no remarkable pathological findings” in the histopathological examination. Tissue samples were stored in paraffin blocks. Fresh 4-μm-thick sections were prepared routinely and stained with hematoxylin-eosin. Histopathological examination was performed under light microscopy by the authors (SH, IK) and tissues were confirmed as being nonneoplastic (“no remarkable findings”).

### MCT Samples

Tissue samples from canine cutaneous MCTs (*n* = 63) submitted for routine analysis to the University of Helsinki, Faculty of Veterinary Medicine, Pathology Unit, in years 2006 to 2017 were used in this study. Cases were selected by screening the same patient data system as for the nonneoplastic specimens, by the inclusion criteria of cutaneous MCT diagnosis in the histopathological examination. MCT diagnoses were confirmed by the authors (SH, IK) by histologic review of hematoxylin-eosin and toluidine blue-stained, 4-µm sections from formalin-fixed, paraffin-embedded tissue blocks. MCTs were graded to either low-grade or high-grade according to the Kiupel 2-tier system.^19^

#### Negative control neoplastic tissues

Round cell tumors that may represent a differential diagnosis for poorly granulated MCTs were selected to study the specificity of CPA3. Biopsy cases for histiocytomas (*n* = 2), plasma cell tumors (*n* = 3), and lymphoma (*n* = 2) were obtained from the tissue archives of the University of Helsinki, Faculty of Veterinary Medicine, Pathology Unit. All tumors were restricted to skin except one oral plasma cell tumor and a splenic follicular lymphoma.

#### Western blot analysis

For Western blot analysis to evaluate antibody specificity, a fresh frozen MCT tumor sample kindly provided by Doctor Piia Marttinen (Veterinary Clinic Apex, Evidensia, Helsinki, Finland), canine muscle tissue obtained from routine necropsy and a human MC cell line (HMC1.1), kindly provided by Professor Gunnar Nilsson (Immunology and Allergy Unit, Department of Medicine Solna, Karolinska Institutet, and Karolinska University Hospital, Stockholm, Sweden), were homogenized by grinding with sand and steel balls in TissueLyser II bead mill (QiaGEN Ltd), suspended in phosphate-buffered saline, and run on 15% SDS-PAGE under reducing conditions. Proteins were electro-transferred onto nitrocellulose membrane (Hybond-C Extra, Amersham). After blocking in 3% defatted milk, the membranes were incubated for 1 hour with rabbit anti-human CPA3 antibody (polyclonal, 1:500, Atlas Antibodies, HPA008689, Sigma-Aldrich). The membranes were then incubated for 1 hour with IRDye 800CW fluorescent goat-anti-rabbit IgG antibody diluted 1:10000 (Li-Cor BioSciences). Immune complexes were visualized with Odyssey FLc digital imaging system (Li-Cor BioSciences).

### Immunohistochemistry

Four-mirometer-thick sections were obtained from the formalin-fixed paraffin blocks of each sample. Slides were deparaffinized, rehydrated in an alcohol series and rinsed in distilled water. Heat-induced antigen retrieval was performed in a water bath (Lab Vision PT Module, Thermo Fisher) using a 10 mM citrate buffer (pH 6) for 20 minutes at 99 °C. Endogenous peroxidase was blocked by immersion in 3% hydrogen peroxide for 10 minutes, and protein blockage was performed by using phosphate buffered saline with 10% bovine serum albumin. Primary antibodies, rabbit anti-human CD117 (polyclonal, 1:600, Agilent, A450229-2) for nonneoplastic tissues and selected MCTs, and rabbit anti-human CPA3 (polyclonal, 1:500, Atlas Antibodies, HPA008689, Sigma-Aldrich) for all tissues, were incubated at room temperature for 60 minutes. Prior the secondary antibody a post-antibody blocking solution was applied (ImmunoLogic). Secondary antibody (goat anti-mouse/rabbit IgG, HRP linked, ImmunoLogic) was incubated in a humid chamber for 30 minutes in room temperature. The immunoreaction was visualized with the Bright DAB Substrate kit (ImmunoLogic) and counterstained with Harris hematoxylin. Sections from canine MCT known to express c-kit were used as positive controls. Negative labeling was assessed in a 2-fold manner. Appearance of any unspecific binding of the primary antibody was evaluated by using rabbit IgG, polyclonal isotype control (1:500, Abcam, ab37415) instead of the primary antibodies. Unspecific binding of the secondary antibody was assessed by omitting the primary antibodies and using instead the antibody diluent buffer (Normal Antibody Diluent, ImmunoLogic). In both cases, the secondary antibody was applied as described above.

Evaluation of CPA3 labeling pattern in MCTs was evaluated from 10 randomly selected high-power fields (HPFs) per section excluding the margins to avoid any possible artefactual staining, and the labeling patterns for CPA3 were recorded. Labeling pattern was considered predominant when found in the majority (>60% to 70%) of neoplastic cells evaluated from the 10 HPFs throughout the section.

### Statistical Methods

Statistical analysis was performed using SPSS v.25 (IBM Corp). Differences were considered significant when *P* < .05. Statistical evaluations of differences between 2 groups were identified using the unpaired Student’s *t* test followed by Mann-Whitney’s *U* test. When appropriate Pearson’s χ^2^ test was used.

## Results

### CPA3 Immunohistochemistry in Nonneoplastic Mast Cells

For the polyclonal antibody against human CPA3 used for this study, the antigen amino acid sequence provided by the manufacturer was predicted to be 86% identical^26^ to the amino acid sequence of canine CPA3 (XM_038432620.1: mast cell carboxypeptidase A [Canis lupus familiaris]). Similarly, the human CPA3 amino acid sequence (AAH12613: carboxypeptidase A3 (mast cell) [Homo sapiens]) and canine sequences were predicted to be 88% identical,^26^ suggesting that the antibody against human CPA3 would cross-react with the canine CPA3 protein (Suppl. Data 1 and 2). In Western blot analysis, the CPA3 antibody marked one strong band from the MCTs and human mast cell homogenates but no signal was detected in muscle tissue (Suppl. Fig. S1). The band in both human mast cell and canine MCT homogenates had a similar molecular weight of approximately 75 kDa. While the canine CPA3 has not been fully sequenced, based on the predicted amino acid sequence, the expected molecular weight was 48.7 kDa (https://web.expasy.org/protparam/). The higher observed molecular weight could represent the CPA3 binding to the negatively charged proteoglycans^34^ or be the result of post-translational modifications.

**Figure 1.**
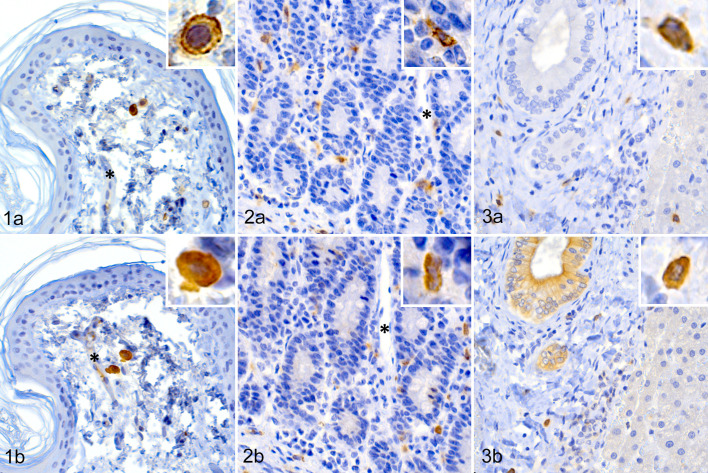
Normal skin, dog. Round cells with a typical mast cell morphology in the dermis are immunolabeled for both c-kit (a) and CPA3 (b). CPA3 labels the cytoplasm diffusely and obscures the nucleus. Mast cells are close to a dermal blood vessel (*). Inset: Higher magnification of a labeled cell. **Figure 2.** Normal small intestine, dog. Numerous c-kit (a) and CPA3 (b) positive cells are present in the lamina propria. The cytoplasm of mast cells is diffusely labeled for CPA3. Mast cells are close to a mucosal blood vessel (*). Inset: Higher magnification of a labeled cell. **Figure 3.** Normal liver, dog. Mast cells in portal areas are immunolabeled for both c-kit (a) and CPA3 (b). CPA3 labels the mast cell cytoplasm diffusely, sometimes condensing to a more dotted pattern. Inset: Higher magnification of a labeled cell.

CPA3 immunoreactivity was detected in round cells with a typical mast cell morphology in all the studied normal nonneoplastic tissues (20/20) and similar cells were also immunopositive for c-kit (Figs. 1 [Fig fig2-03009858211062636] [Fig fig3-03009858211062636]–4). In the skin samples (*n* = 5), scattered c-kit (Fig. 1a) and CPA3 (Fig. 1b) positive cells were observed in the superficial dermis and surrounding the blood vessels. CPA3 immunolabeling was diffuse, intense, and localized to the cytoplasm sometimes obscuring the nucleus. In small intestine (*n* = 5), numerous c-kit (Fig. 2a) and CPA3 (Fig. 2b) positive cells were observed in the lamina propria. These cells were distributed across the lamina propria but were slightly more frequent in the near vicinity of blood vessels. Scattered immunopositive cells were also observed in submucosa. Similar to skin, CPA3 immunoreactivity was observed diffusely in the cytoplasm of the cells, but in some cases condensed to a more dotted pattern.

In the liver samples (*n* = 5), c-kit (Fig. 3a) and CPA3 (Fig. 3b) immunoreactive cells were most numerous around blood vessels, especially in portal areas, and CPA3 immunolabeling was detected diffusely in the cytoplasm with moderate intensity and sometimes with a dotted pattern. Of note, in the liver along the sinusoids, c-kit and a few CPA3 immunopositive cells with a more irregular cytoplasm were also observed. The unusual localization and morphology suggest that these cells could be of macrophage lineage instead of being mast cells. In the liver, some unspecific extracellular CPA3 labeling was also observed in sinusoids, in the lumen of blood vessels and in the epithelial cells of bile ductules.

**Figure 4. fig2-03009858211062636:**
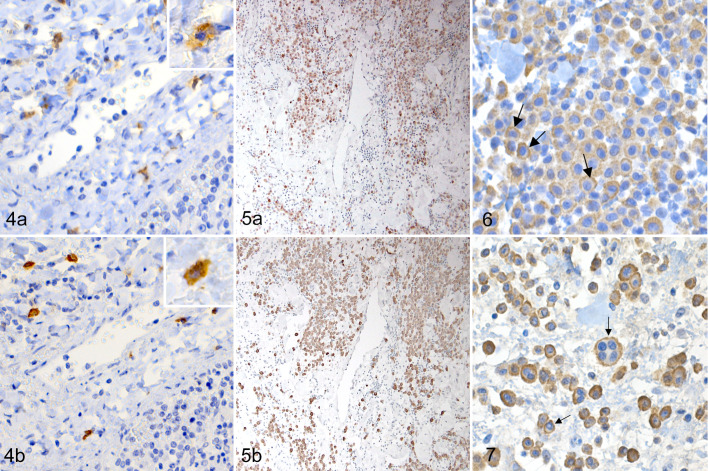
Normal spleen, dog. Both c-kit (a) and CPA3 (b) positive cells are in the white pulp. Inset: higher magnification of a labeled cell. **Figure 5**. High-grade MCT, skin, dog. Similar cells are labeled with antibodies to c-kit (a) and to CPA3 (b). **Figure 6**. Low-grade MCT, skin, dog. Membranous CPA3 labeling pattern (arrows) is characterized by membrane-associated labeling with weak or no labeling in the cytoplasm surrounding the nucleus. **Figure 7.** High-grade MCT, skin, dog. Membrane-associated labeling (arrows) is present in neoplastic cells, including within the multinucleated neoplastic cell.

In the splenic samples (*n* = 5), c-kit (Fig. 4a) and CPA3 (Fig. 4b) immunoreactive cells were found in the white pulp and in areas surrounding white pulp.

### MCT Sample Population

Sixty-three MCT samples from 56 dogs were included in this study (Suppl. Tables S2 and S3). By the Kiupel 2-tier system,^19^ 53/63 (84%) were low-grade and 10/63 (16%) high-grade MCTs. MCT samples from the 56 dogs represented 28 different dog breeds and 3 mixed breed dogs (breed of 1 dog was unknown). Golden Retriever, Labrador Retriever, and Boxer were overrepresented in our material (11%, 9%, and 9%, respectively). Median age was 8 years. In 12 cases, there was no information about the age of the dog. The median age for low-grade and high-grade MCTs was 7.8 years and 10.9 years, respectively. Among the total population, 25/56 (45%) dogs were males, of which 9 (36%) were neutered; 29/56 (52%) were females, of which 15 (52%) were spayed. In 2 cases, there was no information about the sex of the dog. Median body weight in the study population was 26.1 kg (range 6.6–51.1 kg). In the low-grade MCT group, median weight was higher (27.8 kg, range 6.6–51.1 kg) than in the high-grade MCT group (19.4 kg, range 8.3–37.6 kg). The most common site for a MCT was the limb or flank followed by head/neck, abdominal, and tail/perineal skin. In 4 cases there was no information about the tumor location. Tumor location, sex, or weight was not associated with MCT grades.

### Immunoreactivity of Canine Cutaneous MCTs for CPA3

CPA3 was expressed in all 63/63 cutaneous MCTs. No immunoreactivity was detected in the other canine round cell tumors, irrespective of their location (whether cutaneous, mucosal, or visceral; Suppl. Figs. S2–S6). Furthermore, no unspecific staining was observed in the mast cells of the studied MCT samples using the rabbit IgG, polyclonal isotype as a negative control (Suppl. Fig. S7).

To see whether CPA3 labeling was comparable to the most commonly used MCT marker c-kit, we performed c-kit and CPA3 immunolabeling in serial sections from 3 tumor samples (Fig. 5a, b). In the samples, similar cells were labeled and in most cases similar labeling patterns were found with both markers. However, focal cytoplasmic labeling of c-kit did not always translate to focal cytoplasmic labeling of CPA3.

Protein expression was localized to cytoplasm or near the cell membrane. In the MCTs, 3 distinct labeling patterns were recorded: membranous, diffuse cytoplasmic, and focal cytoplasmic. All tumors had multiple CPA3 labeling patterns, most commonly both membranous and diffuse cytoplasmic. Membranous labeling was present in 63/63 (100%) MCT samples irrespective of tumor grading and was characterized by tightly membrane-bound ring-like labeling, leaving the cytoplasm fully unlabeled (Figs. 6, 7). Membranous labeling was the most predominant pattern in 7/53 (13%) low-grade tumors and in 1/10 (10%) high-grade tumors.

**Figure 8. fig3-03009858211062636:**
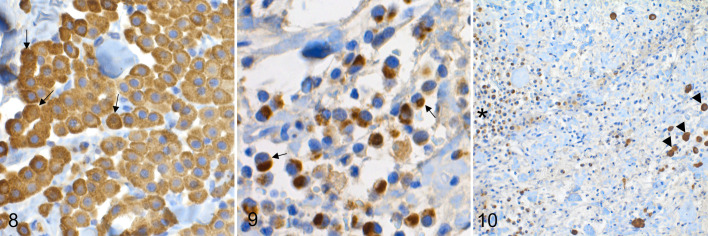
Low-grade MCT, skin, dog. Diffuse cytoplasmic CPA3 labeling pattern (arrows) is characterized by evenly stained cytoplasm, sometimes obscuring the nucleus. A membranous pattern is also present. **Figure 9**. High-grade MCT, skin dog. The focal cytoplasmic CPA3 labeling pattern (arrows) is characterized by focal intensively labeled stipples, spots, or dots in the cytoplasm. The cytoplasmic CPA3-positive aggregates vary in size, and in these cells the nucleus is often eccentric. **Figure 10**. High-grade MCT, skin, dog. Neoplastic cells in the main tumor cell mass (black asterisk) label weakly or moderately for CPA3 with mostly focal cytoplasmic labeling pattern. The infiltrating mast cells (arrow heads) label more intensely and diffusely for CPA3.

Diffuse cytoplasmic labeling was also present in all 63/63 (100%) MCT samples. Diffuse labeling varied in intensity but in all samples filled the cytoplasm completely and sometimes, in the most intensely stained cells, obscured the nucleus (Fig. 8). Diffuse cytoplasmic labeling was the most predominant pattern within the tumor in 46/53 (87%) low-grade tumors and in 9/10 (90%) high-grade tumors.

None of the tumors had focal cytoplasmic labeling as the predominant labeling pattern, and overall this labeling was the least common labeling pattern observed in the tumors. Yet, focal cytoplasmic labeling was significantly more common (*P* = .001) in the high-grade tumors 7/10 (70%) than in low-grade tumors 8/53 (15%). Focal cytoplasmic labeling pattern was characterized by varying sized, round to oval, dotted/stippled accumulations of cytoplasmic protein often eccentrically, with the nucleus on the opposite side of the cell (Fig. 9).

The CPA3 labeling intensity varied between and within individual samples. In the low-grade MTCs, the intensity was strong or moderate almost throughout the sample, whereas in the high-grade MCTs the intensity was sometimes weaker, yet in each sample at least a part of the sample was strongly labeled. Nonneoplastic mast cells were commonly more intensely labeled than the neoplastic mast cells, especially in the high-grade tumors (Fig. 10).

## Discussion

Mast cells are pleiotropic inflammatory cells with variety of physiological and pathological functions. The effector functions of mast cells are dispatched by the mediators released by exocytosis from the activated cells. The neutral granule proteases constitute between 30% and 50% of the total protein content of mast cells, and thus under mast cell–activating conditions these enzymes represent, in terms of mass, the major group of mediators released by exocytosis.^12^ The granule proteases fall into 3 classes, tryptases, chymases, and CPA3. Here we describe CPA3 expression in canine mast cells and in MCTs.

In both nonneoplastic and neoplastic mast cells the protein expression was restricted to cytoplasm as expected based on previous knowledge on CPA3 protein (Human Protein Atlas, https://www.proteinatlas.org/ENSG00000163751-CPA3/cell).^45^ In the nonneoplastic mast cells, CPA3 was expressed in all the studied tissues including the skin, small intestine (both mucosa and submucosa), liver, and spleen, thus covering mast cells of mucosal and connective tissues. In humans and in rodents, CPA3 expression is more limited, with no expression in mucosal mast cells.^5,34,47^ We also observed scattered CPA3 and c-kit immunopositive cells in the liver that were deemed to be of macrophage lineage instead of mast cells and such c-kit expressing macrophages have been previously reported in liver.^17,27^

In addition to nonneoplastic mast cells, CPA3 was also expressed in all the studied cutaneous MCTs with a strong labeling intensity. Although a full comparison of c-kit and CPA3 labeling was not performed in all of the MCTs, CPA3 immunoreactivity was comparable to that of c-kit protein expression in both the tissue-resident mast cells and in the selected MCTs studied.

Like almost all immunohistochemistry markers, c-kit is not fully specific as its expression has been reported also in tumors other than MCTs. Of canine cutaneous round cell malignancies, c-kit expression is common in dermal melanocytomas but less frequent in more aggressive mucosal or submucosal melanomas.^30^ Similarly, c-kit expression has been reported in a cutaneous epitheliotropic lymphoma.^40^ In other than round cell tumors, c-kit is also commonly expressed in seminomas and in seminomatous components of mixed testicular tumors.^16^ Since the expression of CPA3 is restricted to mast cells,^34^ and no positive immunolabeling for CPA3 was detected in the other round cell tumors (lymphoma, histiocytoma, and plasma cell tumor) studied here, CPA3 could be used as an additional marker alongside c-kit to differentiate poorly differentiated round cell tumors. The additional validation experiments in this study (WB analysis, bioinformatics-based approach, selection of appropriate positive and negative controls) further support CPA3 as marker for mast cells in the dog. There are previously published guidelines for the validation of novel immunohistochemistry markers,^45^ and where applicable, we have adhered to these guidelines. Yet further studies could highlight the possible CPA3 expression also in other canine malignancies and to compare its specificity to c-kit.

The true biological role of CPA3 remains elusive, yet some clues exist linking CPA3 expression to tumor progression. One of the identified substrates of CPA3 is angiotensin I (Ang1). CPA3 cleaves a single amino acid from the 10 amino acid long Ang1, creating a c-terminally truncated bioactive Ang(1–9) polypeptide.^13,22^ In vitro, it has been shown that Ang(1–9) can induce cell proliferation, promote cell motility, and produce a pro-tumorigenic gene signature in prostate epithelial cancer cells.^6^ Furthermore, progressively increased expression of CPA3, together with chymase and tryptase activities, have been described in a chemically induced mouse epidermal tumor model during tumor progression.^43^ Interestingly, increased CPA3 gene expression has been described in human patients with colorectal cancer nonresponsive to treatment.^4^ Furthermore, patients with oral squamous cell carcinoma with extracapsular spread and worse overall survival harbor a unique, predictive 11 gene signature, one of the genes being CPA3.^44^

In the present study, we described 3 distinct labeling patterns for CPA3 in cutaneous MCTs. The most common labeling patterns were membranous and diffuse, which were present in all of the studied MCTs, while focal cytoplasmic was the least common pattern and was significantly more frequent in the high-grade tumors. It could be hypothesized that the focal labeling pattern would reflect an altered protein expression in these more aggressive tumors, and it would be interesting to evaluate the outcomes of those dogs with a low-grade MCT and the focal cytoplasmic labeling pattern, to see whether this pattern precedes a shift to a more aggressive tumor phenotype. Yet any conclusions of CPA3 as a prognostic marker cannot be evaluated from the present data since no follow-up data of the dogs was available.

CPA3 in mast cells is tightly bound to proteoglycans in the secretory granules expressed by the different mast cell phenotypes.^33^ It has been shown that less differentiated and hence more biologically aggressive MCT have fewer secretory granules.^41^ This could be another explanation for the seemingly reduced protein expression as detected in the dotted, more scattered labeling in the focal cytoplasmic labeling pattern, present more frequently in the high-grade tumors. CPA3 labeling intensity also varied between and within the samples irrespective of the labeling pattern documented. The variation in CPA3-labeling intensity was also observed in the toluidine blue–stained sections used to conform the MCT diagnosis. This could further indicate that positive immunolabeling for CPA3 could correspond to the granularity of the mast cells.

Overall, our findings indicate that CPA3 expression and the pattern of immunolabeling in MCTs can provide a novel, additional biomarker for differentiating these common canine tumors.

## Supplemental Material

Supplemental Material, sj-pdf-1-vet-10.1177_03009858211062636 - Carboxypeptidase A3 expression in canine mast cell tumors and tissue-resident mast cells Click here for additional data file. Supplemental Material, sj-pdf-1-vet-10.1177_03009858211062636 for Carboxypeptidase A3 expression in canine mast cell tumors and tissue-resident mast cells by Sanna Hämäläinen, Lauri Kareinen, Antti Sukura and Ilona Kareinen in Veterinary Pathology
