# Supplementary material for: Carboxypeptidase A3 expression in canine mast cell tumors and tissue-resident mast cells
Source: Vet Pathol. 2021 Dec 12;59(2):236–43. doi: 10.1177/03009858211062636 (PMC8928232; doi:10.1177/03009858211062636)
Supplement: Supplemental Material, sj-pdf-1-vet-10.1177_03009858211062636 - Carboxypeptidase A3 expression in canine mast cell tumors and tissue-resident mast cells [file sj-pdf-1-vet-10.1177_03009858211062636.pdf]

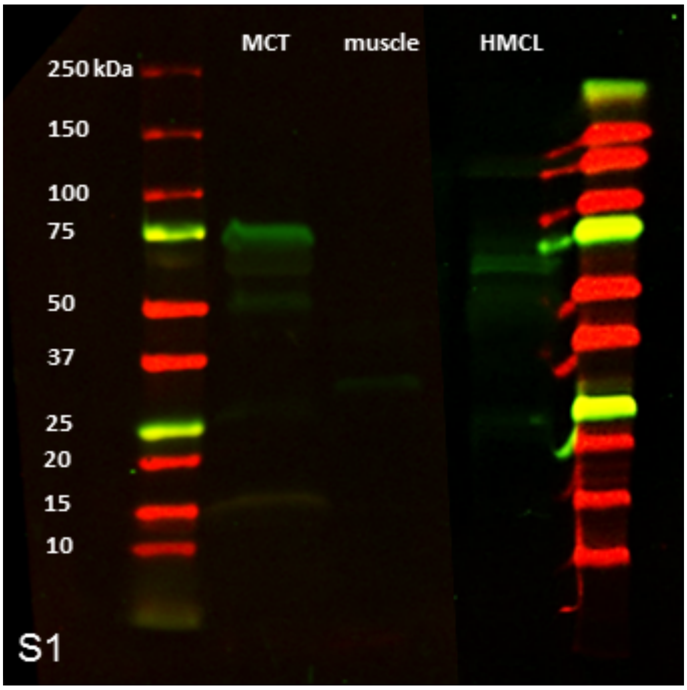

Supplemental Figure S1. Western blot analysis to detect CPA3 protein from canine MCT and human MC lysate (HMCL). Canine muscle tissue was used as a negative control tissue. The tissues were homogenized and probed with the rabbit anti-human CPA3 antibody. The antibody was able to recognize a band of approximately 75 kDa in size from both canine and human MC lysates while no bands were detected in mucle tissue.

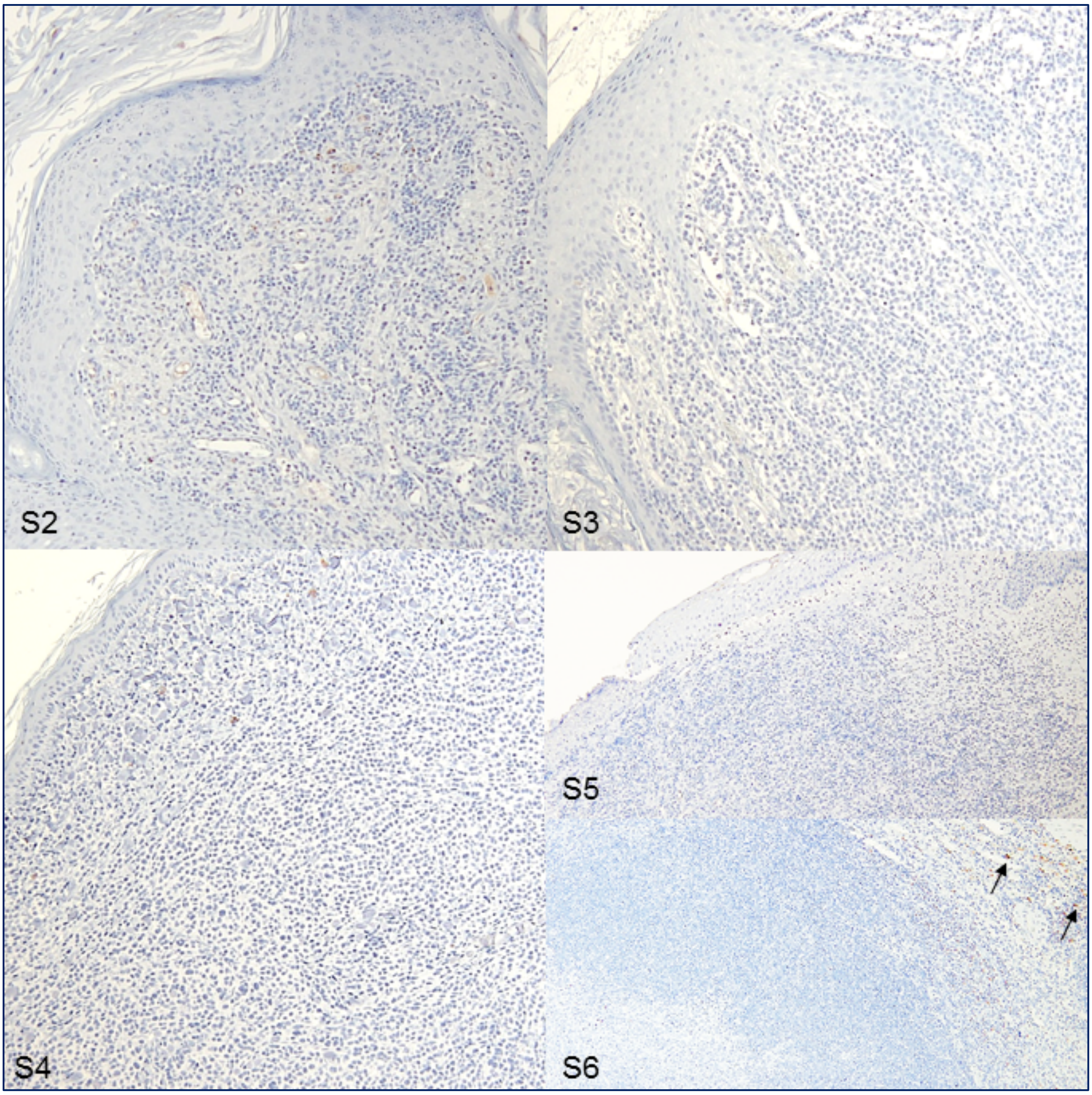

Supplemental Figures S2-S6. CPA3 immunoreactivity in selected canine round cell tumors, skin, dog. No CPA3 positive neoplastic cells were observed in cutaneous lymphoma (**S2**), histiocytoma (**S3**) or plasmacytoma (**S4**). Furthermore, no immunoreactivity was detected in oral plasma cell tumor (**S5**) or in the splenic follicular lymphoma (**S6**). Few cells outside the tumor mass stain positive in the splenic sample (**S6**, arrows), but similar cells were positive also with the c-kit antibody and the negative rabbit IgG polyclonal isotype control stain.

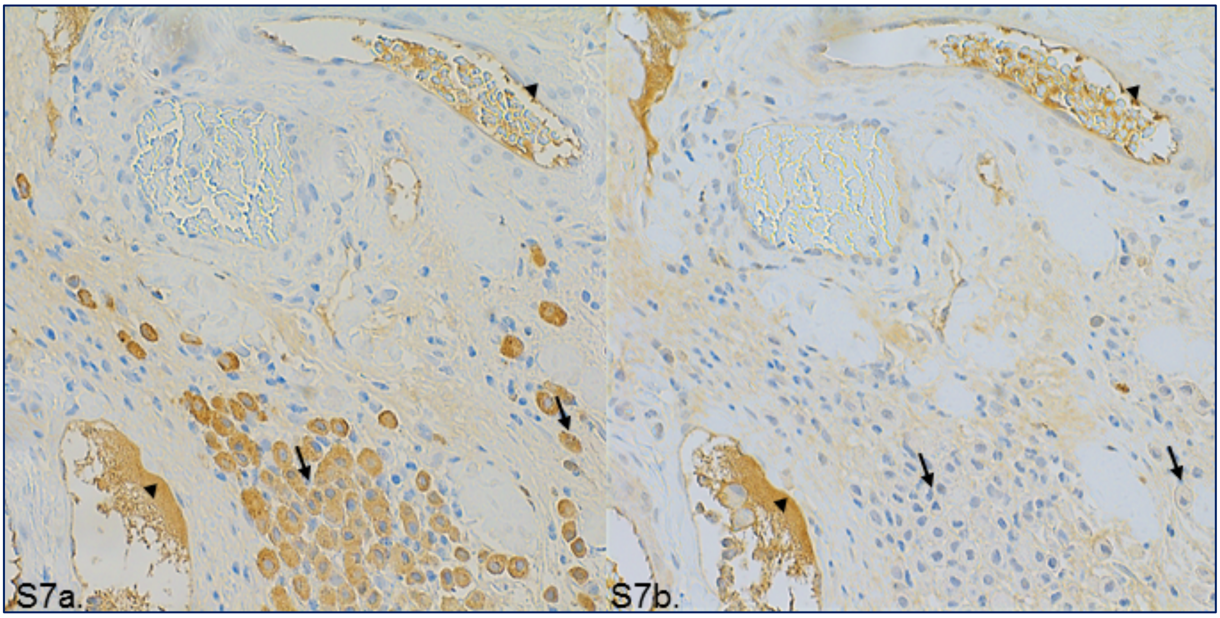

Supplemental Figure S7. The unspecific binding of the primary antibody was evaluated using rabbit IgG, polyclonal isotype control, skin, dog. The micrographs show representative positive (a) and negative (b) staining of a high-grade MCT from a female flat-coated retriever. MCs (arrows) in the MCT were stained positively with the rabbit anti-human CPA3 antibody (a), while no MCs were stained using the rabbit IgG, polyclonal isotype control (b). Unspecific intraluminal and intimal staining was observed in the blood vessels (arrowheads) using both the CPA3 antibody and the rabbit IgG, polyclonal isotype control.

Supplemental Table S1. Non-neoplastic tissues for CPA analysis.

	Breed	Age (years)	Sex	Weight (kg)	Tissues analyzed
Dog 1	Rottweiler	6	M	41	Skin
Dog 2	Smooth fox terrier	4	F	7.3	Skin
Dog 3*	American Cocker Spaniel	8	F	9.5	Skin
Dog 4	Finnish Lapphund	7	F,N	15	Skin
Dog 5	Spanish Water Dog	8	M,N	29	Skin
Dog 6	Labrador retreiver	1	F	17	Sp, Li, SI
Dog 7	French water dog	7	F	17	Sp, Li, SI
Dog 8	Miniature Schnauzer	0.2	F	3.8	Sp, Li, SI
Dog 9	Greyhound	2	F	26	Sp, Li, SI
Dog 10	Bordercollie	1	M	21	Sp, Li
Dog 11	Boxer	1	F	23	SI

Non-neoplastic canine MCs were evaluated for their CPA tissue expression by immunohistochemistry from cutaneous, hepatic, splenic and small intestinal tissues. Sample marked with an asterix denotes for a biopsy sample, other samples were obtained from autopsies. F, female; Li, Liver; M, male; N, neutered; SI, small intestine; Sp, Spleen.

Supplemental Table S2. Patient data of dogs with cutaneous low grade or high grade MCTs

			Low grade (n=53)	High grade (n=10)
			Median (range)	
**Age**			7.8 y (3 m – 13 y)	10.9 y (6 – 13.1 y)
**Weight**			27.8 kg (6.6–51.1 kg)	19.4 kg (8.3–37.6 kg)
			n (%)	
**Sex**	**Male**	**Intact**	16 (31)	1 (10)
		**Castrated**	6 (12)	4 (40)
	**Female**	**Intact**	12 (24)	3 (30)
		**Spayed**	17 (33)	2 (20)
**Total***			51 (100)	10 (100)
**Location**		**Limb**	14 (29)	4 (40)
		**Flank**	10 (20)	1 (10)
		**Head/neck**	11 (22)	3 (30)
		**Abdominal skin**	7 (14)	1 (10)
		**Tail/perineal skin**	7 (14)	1 (10)
**Total†**			49 (100)	10 (100)

*Gender was not recorded for 2 and †location for 4 low grade MCTs.

Supplemental Table S3. Breeds of the sample population.

**Breed**	**n**	**%**
Golden Retriever	6	10.9
Boxer	5	9.1
Labrador Retriever	5	9.1
Boston Terrier	3	5.5
Dogo Argentino	3	5.5
Mixed breed	3	5.5
Australian Terrier	2	3.6
Irish Terrier	2	3.6
Fox Terrier	2	3.6
Nova Scotia Duck Tolling Retriever	2	3.6
French Bulldog	2	3.6
German Shepherd	2	3.6
Flat-coated Retriever	2	3.6
Staffordshire Bullterrier	2	3.6
American Cocker Spaniel	1	1.8
Bernese Mountain Dog	1	1.8
Brasilian Terrier	1	1.8
Doberman	1	1.8
Spanish Water Dog	1	1.8
Jack Russell Terrier	1	1.8
Miniature Pinscher	1	1.8
Pug	1	1.8
Parson Russell Terrier	1	1.8
Petit Brabancon	1	1.8
Rhodesian Ridgeback	1	1.8
Shetland Sheepdog	1	1.8
Finnish Lapphund	1	1.8
Whippet	1	1.8
Total	55	100

63 MCT samples from 56 dogs were included in this study. The breed of one of the dogs was unknown.

Supplemental Data 1. Nucleotide alignment of human and canine CPA3 sequences generated with the BLAST tool[1].

```
Query: Homo sapiens carboxypeptidase A3 (CPA3), mRNA Query ID: NM_001870.4 Length: 1762

Sbjct: Canis lupus familiaris carboxypeptidase A3 (CPA3), mRNA
Sequence ID: XM_038571158.1 Length: 1693
Range 1: 64 to 1679

Score:1903 bits(1030), Expect:0.0,
Identities:1442/1639(88%),  Gaps:36/1639(2%), Strand: Plus/Plus

Query  1      CAAAGAAGAACCATGAGGCTCATCCTGCCTGTGGGTTTGATTGCTACCACTCTTGCAATT  60
              ||||||||||||||| || ||||||||||||||||| |||| |||||||| |||||||||
Sbjct  64     CAAAGAAGAACCATGTGGTTCATCCTGCCTGTGGGTCTGATCGCTACCACCCTTGCAATT  123

Query  61     GCTCCTGTCCGCTTTGACAGGGAGAAGGTGTTCCGCGTGAAGCCCCAGGATGAAAAACAA  120
              ||||||||||||||||||||||||||||| ||||||||||||||||||||||||||||||
Sbjct  124    GCTCCTGTCCGCTTTGACAGGGAGAAGGTATTCCGCGTGAAGCCCCAGGATGAAAAACAA  183

Query  121    GCAGACATCATAAAGGACTTGGCCAAAACCAATGAGCTTGACTTCTGGTATCCAGGTGCC  180
              ||| | ||||||||||||||||||||||||||  ||||||||||||||||||||| ||||
Sbjct  184    GCAAATATCATAAAGGACTTGGCCAAAACCAACCAGCTTGACTTCTGGTATCCAGATGCC  243

Query  181    ACCCACCACGTAGCTGCTAATATGATGGTGGATTTCCGAGTTAGTGAGAAGGAATCCCAA  240
              |||||||||||| | |||||||||| | | ||||||| ||||||||| ||||||||||| 
Sbjct  244    ACCCACCACGTAACCGCTAATATGACGATAGATTTCCAAGTTAGTGAAAAGGAATCCCAG  303

Query  241    GCCATCCAGTCTGCCTTGGATCAAAATAAAATGCACTATGAAATCTTGATTCATGATCTA  300
               ||||||||||||||||||| |||||||| ||||||||||||||||||||||||||||||
Sbjct  304    TCCATCCAGTCTGCCTTGGACCAAAATAATATGCACTATGAAATCTTGATTCATGATCTA  363

Query  301    CAAGAAGAGATTGAGAAACAGTTTGATGTTAAAGAAGATATCCCAGGCAGGCACAGCTAC  360
              ||||||||||||||||||||||||||||||||||||||||||||||||||||||||||| 
Sbjct  364    CAAGAAGAGATTGAGAAACAGTTTGATGTTAAAGAAGATATCCCAGGCAGGCACAGCTAT  423

Query  361    GCAAAATACAATAATTGGGAAAAGATTGTGGCTTGGACTGAAAAGATGATGGATAAGTAT  420
              |||||||| ||||| ||||| |||||||| |||||||||||||| ||| |   ||||| ||
Sbjct  424    GCAAAATATAATAACTGGGACAAGATTGTTGCTTGGACTGAAAAAATGGTTCATAAGCAT  483

Query  421    CCTGAAATGGTCTCTCGTATTAAAATTGGATCTACTGTTGAAGATAATCCACTATATGTT  480
              ||||| |||||||||||||||||||||||| | |||||||||||||||||||||||||||
Sbjct  484    CCTGATATGGTCTCTCGTATTAAAATTGGAACCACTGTTGAAGATAATCCACTATATGTT  543

Query  481    CTGAAGATTGGGGAAAAGAATGAAAGAAGAAAGGCTATTTTTACGGATTGTGGCATTCAC  540
              || ||| ||||| ||  | |||||||||||||||||||||||| ||||||||||||||| 
Sbjct  544    CTCAAGGTTGGGAAAGGGGATGAAAGAAGAAAGGCTATTTTTATGGATTGTGGCATTCAT  603

Query  541    GCACGAGAATGGGTCTCCCCAGCATTCTGCCAGTGGTTTGTCTATCAGGCAACCAAAACT  600
              |||||||||||| ||||||||||||| |||||||||||||||||||||||||||||||| 
Sbjct  604    GCACGAGAATGGATCTCCCCAGCATTTTGCCAGTGGTTTGTCTATCAGGCAACCAAAACC  663

Query  601    TATGGGAGAAACAAAATTATGACCAAACTCTTGGACCGAATGAATTTTTACATTCTTCCT  660
              ||||| | ||| |||||||||||||||||| |||||||||||||||||||  ||||||||
Sbjct  664    TATGGAAAAAATAAAATTATGACCAAACTCCTGGACCGAATGAATTTTTATGTTCTTCCT  723

Query  661    GTGTTCAATGTTGATGGATATATTTGGTCATGGACAAAGAACCGCATGTGGAGAAAAAAT  720
              || |||||||||||||||||||||||||||||||| |||||||||||||||||||||||
Sbjct  724    GTATTCAATGTTGATGGATATATTTGGTCATGGACACAGAACCGCATGTGGAGAAAAAAT  783

Query  721    CGTTCCAAGAACCAAAACTCCAAATGCATCGGCACTGACCTCAACAGGAATTTTAATGCT  780
              ||||||||||||||||||||||||||||| |||||||||||||||||||| |||||||||
Sbjct  784    CGTTCCAAGAACCAAAACTCCAAATGCATTGGCACTGACCTCAACAGGAACTTTAATGCT  843

Query  781    TCATGGAACTCCATTCCTAACACCAATGACCCATGTGCAGATAACTATCGGGGCTCTGCA  840
```

```
             || ||||||||  |||  ||||||||  | ||||||||| |||  ||||||||| |   |
Sbjct  844   TCGTGGAACTCTTTTCAGAACACCAAGAATCCATGTGCAAATATATATCGGGGCCCAAAA  903

Query  841   CCAGAGTCCGAGAAAGAGACGAAAGCTGTCACTAATTTCATTAGAAGCCACCTGAATGAA  900
             |||||||| ||||||||||| |||||||||||| | |||||  ||||||| |||||   |
Sbjct  904   CCAGAGTCTGAGAAAGAGACCAAAGCTGTCACTGACTTCATACGAAGCCATCTGAAATCA  963

Query  901   ATCAAGGTTTACATCACCTTCCATTCCTACTCCCAGATGCTATTGTTTCCCTATGGATAT  960
             |||||||  || ||||||||||| || || ||||||||||||||||||||||||||||||
Sbjct  964   ATCAAGGCCTATATCACCTTCCACTCTTATTCCCAGATGCTATTGTTTCCCTATGGATAT  1023

Query  961   ACATCAAAACTGCCACCTAACCATGAGGACTTGGCCAAAGTTGCAAAGATTGGCACTGAT  1020
             ||||||||||||||| |||||||||||||| ||| ||||||||||||||| ||||| |||
Sbjct  1024  ACATCAAAACTGCCATCTAACCATGAGGACCTGGACAAAGTTGCAAAGATCGGCACAGAT  1083

Query  1021  GTTCTATCAACTCGATATGAAACCCGCTACATCTATGGCCCAATAGAATCAACAATTTAC  1080
             |||||| ||||||||||||||||||||||||||||||||||||||| ||||||  | |||
Sbjct  1084  GTTCTAGCAACTCGATATGAAACCCGCTACATCTATGGCCCAATAGCATCAACTTTCTAC  1143

Query  1081  CCGATATCAGGTTCTTCTTTAGACTGGGCTTATGACCTGGGCATCAAACACACATTTGCC  1140
             |||| |||||||||||||||||||||||||||  || |||||||||||||||||||||||
Sbjct  1144  CCGACATCAGGTTCTTCTTTAGACTGGGCTTACAACGTGGGCATCAAACACACATTTGCC  1203

Query  1141  TTTGAGCTCCGAGATAAAGGCAAATTTGGTTTTCTCCTTCCAGAATCCCGGATAAAGCCA  1200
             ||||| |||||||||||||||||||  |||||||||||||||||||| | ||||||| ||
Sbjct  1204  TTTGAACTCCGAGATAAAGGCAAATACGGTTTTCTCCTTCCAGAATCTCTGATAAAGTCA  1263

Query  1201  ACGTGCAGAGAGACCATGCTAGCTGTCAAATTTATTGCCAAGTATATCCTCAAGCATACT  1260
             || || | ||| ||| |||||||||| |||||||||||||| ||||| ||||||||||||
Sbjct  1264  ACCTGTAAAGAAACCTTGCTAGCTGTTAAATTTATTGCCAAATATATTCTCAAGCATACT  1323

Query  1261  TCCTAAAGAACTGCCCTCTGTTTGGAATAAGCCAATTAATCCTTTTTTGTGCCTTTCATC  1320
             |||||||||| ||  ||| ||| |||||||| ||| ||| |||| ||||||||||| |||
Sbjct  1324  TCCTAAAGAAGTGGACTCAGTTAGGAATAAGTCAAATAACCCTTCTTTGTGCCTTTTATC  1383

Query  1321  AGAAAGTCAATCTTCAGTTATCCCCAAATGCAGCTTCTATTTCACCTGA----ATCCTTC  1376
             |||||||||||||||| ||||||| | ||  |||||| |  | ||||||    |||| ||
Sbjct  1384  AGAAAGTCAATCTTCAATTATCCCTAGATATAGCTTCCACATTACCTGAAGTGATCCCTC  1443

Query  1377  TCTTGCTCATTTAAGTCCCATGTTACTGCTGTTTGCTTTTACTTACTTTCAGTAGCACCA  1436
             ||||||||| ||||||||  | ||  |  | ||| |||  |||| ||  | |||||||
Sbjct  1444  TCTTGCTCACTTAAGTCC--T-TTG-T--T-TTTA-TTT-ACTT--TTA-A-TAGCACCT  1490

Query  1437  TAACGAAGTAGCTTTAAGTGAAACCTTTTAACTACCTTTCTTTGCTCCAAGTGAAGTTTG  1496
             |||| || |||||||||||      ||| ||||||||||||||||||||| | |||||||
Sbjct  1491  TAACAAA-TAGCTTTAAGT------TTTCAACTACCTTTCTTTGCTCCAAATAAAGTTTG  1543

Query  1497  GACCCAGCAGAAAGCATT-A---TTTTGAAAGGTGATATACAGTGGGGCACAGAAAACAA  1552
              |||||| | |||| ||| |   ||||||||  |||||||| ||| | ||||||||| ||
Sbjct  1544  AACCCAGTAAAAAGTATTGAGTGTTTTGAAACATGATATACGGTGAGTCACAGAAAAGAA  1603

Query  1553  ATGAAAACCTTCAGTTTCTC-A----CAGATTTTCACCATGTGGCTTCATCAATTTATGT  1607
             |||| ||  ||||||||||| |    ||| ||||  |||||||||||||||| || ||||
Sbjct  1604  ATGAGAAT-TTCAGTTTCTCCAGAATCAGGTTTT--CCATGTGGCTTCATCATTTCATGT  1660

Query  1608  GCTAATACAATAAAATAAA  1626
              |||||| |||||||||||
Sbjct  1661  ACTAATATAATAAAATAAA  1679
```

Supplemental Data 2. Amino acid alignment of human and canine CPA3 sequences generated with the BLAST tool[1]. CPA3 antibody target epitope is denoted with red.

```
Query: mast cell carboxypeptidase A preproprotein [Homo sapiens] Query ID: NP_001861.2 Length:
417

Sbjct: mast cell carboxypeptidase A [Canis lupus familiaris]
Sequence ID: XP_038427086.1 Length: 417
Range 1: 1 to 417

Score:782 bits(2019), Expect:0.0,
Method:Compositional matrix adjust.,
Identities:366/417(88%), Positives:392/417(94%), Gaps:0/417(0%)

Query  1    MRLILPVGLIATTLAIAPVRFDREKVFRVKPQDEKQADIIKDLAKTNELDFWYPGATHHV  60
            M  ILPVGLIATTLAIAPVRFDREKVFRVKPQDEKQA+IIKDLAKTN+LDFWYP ATHHV
Sbjct  1    MWFILPVGLIATTLAIAPVRFDREKVFRVKPQDEKQANIIKDLAKTNQLDFWYPDATHHV  60

Query  61   AANMMVDFRVSEKESQAIQSALDQNKMHYEILIHDLQEEIEKQFDVKEDIPGRHSYAKYN  120
             ANM +DF+VSEKESQ+IQSALDQN MHYEILIHDLQEEIEKQFDVKEDIPGRHSYAKYN
Sbjct  61   TANMTIDFQVSEKESQSIQSALDQNNMHYEILIHDLQEEIEKQFDVKEDIPGRHSYAKYN  120

Query  121  NWEKIVAWTEKMMDKYPEMVSRIKIGSTVEDNPLYVLKIGEKNERRKAIFTDCGIHAREW  180
            NW+KIVAWTEKM+ K+P+MVSRIKIG+TVEDNPLYVLK+G+ +ERRKAIF DCGIHAREW
Sbjct  121  NWDKIVAWTEKMVHKHPDMVSRIKIGTTVEDNPLYVLKVGKGDERRKAIFMDCGIHAREW  180

Query  181  VSPAFCQWFVYQATKTYGRNKIMTKLLDRMNFYILPVFNVDGYIWSWTKNRMWRKNRSKN  240
            +SPAFCQWFVYQATKTYG+NKIMTKLLDRMNFY+LPVFNVDGYIWSWT+NRMWRKNRSKN
Sbjct  181  ISPAFCQWFVYQATKTYGKNKIMTKLLDRMNFYVLPVFNVDGYIWSWTQNRMWRKNRSKN  240

Query  241  QNSKCIGTDLNRNFNASWNSIPNTNDPCADNYRGSAPESEKETKAVTNFIRSHLNEIKVY  300
            QNSKCIGTDLNRNFNASWNS  NT +PCA+ YRG  PESEKETKAVT+FIRSHL  IK Y
Sbjct  241  QNSKCIGTDLNRNFNASWNSFQNTKNPCANIYRGPKPESEKETKAVTDFIRSHLKSIKAY  300

Query  301  ITFHSYSQMLLFPYGYTSKLPPNHEDLAKVAKIGTDVLSTRYETRYIYGPIESTIYPISG  360
            ITFHSYSQMLLFPYGYTSKLP NHEDL KVAKIGTDVL+TRYETRYIYGPI ST YP SG
Sbjct  301  ITFHSYSQMLLFPYGYTSKLPSNHEDLDKVAKIGTDVLATRYETRYIYGPIASTFYPTSG  360

Query  361  SSLDWAYDLGIKHTFAFELRDKGKFGFLLPESRIKPTCRETMLAVKFIAKYILKHTS  417
            SSLDWAY++GIKHTFAFELRDKGK+GFLLPES IK TC+ET+LAVKFIAKYILKHTS
Sbjct  361  SSLDWAYNVGIKHTFAFELRDKGKYGFLLPESLIKSTCKETLLAVKFIAKYILKHTS  417
```

Reference:

1. Madden T. The BLAST Sequence Analysis Tool. 2003 Aug 13
